# Full BLOOD count TRends for colorectal cAnCer deteCtion (BLOODTRACC): external validation of dynamic clinical prediction models for early detection of colorectal cancer in primary care

**DOI:** 10.1186/s12885-026-16179-9

**Published:** 2026-05-14

**Authors:** Pradeep S. Virdee, Jacqueline Birks, Tim Holt, Kym I.E. Snell, Gary Abel, Brian D. Nicholson

**Affiliations:** 1 Nuffield Department of Primary Care Health Sciences, University of Oxford, Radcliffe Primary Care Building, Radcliffe Observatory Quarter, Woodstock Road, Oxford, OX2 6GG UK; 2 Oxford University Hospital NHS Trust, Oxford, UK; 3 Department of Applied Health Sciences, School of Health Sciences, College of Medicine and Health, University of Birmingham, Birmingham, UK; 4 National Institute for Health and Care Research (NIHR) Birmingham Biomedical Research Centre, Birmingham, UK; 5 University of Exeter Medical School, University of Exeter, Exeter, UK

**Keywords:** Full blood count, Blood test, Primary care, Colorectal cancer, Prediction model, Joint modelling of longitudinal and time-to-event data.

## Abstract

**Background:**

Colorectal cancer has low survival rates when diagnosed late-stage. We previously developed sex-specific dynamic risk prediction models utilising trends in the full blood count (FBC), a blood test commonly performed in primary care, to support early detection. We aimed to externally validate these prediction models.

**Methods:**

We performed a cohort study of patients with at least one haemoglobin, mean cell volume, and platelet test. Patients were aged at least 40 years at their current test and had no history of colorectal cancer. The models included age (years) at current test and simultaneous trends over historical tests measured over five years before the current test to inform two-year risk of colorectal cancer diagnosis. Performance measures included the c-statistic and calibration slope.

**Results:**

We included 2,956,977 males and 3,561,349 females, with 0.4% (*n* = 12,578) and 0.3% (*n* = 11,939) diagnosed with colorectal cancer, respectively. The c-statistic (95% CI) was 0.73 (0.72–0.73) for males and 0.74 (0.74–0.75) for females. The calibration slope (95% CI) was 0.92 (0.89–0.94) for males and 0.95 (0.93–0.98) for females. Calibration was good in subgroups of patient data, except under-predicted risk in those aged 70 + years, White individuals, and those with higher IMD. The c-statistic (95% CI) was similar regardless of the number of repeat tests used to define trend and increased as the longitudinal trend window increased until around 2.5-3.0 years for men (0.73 (0.71–0.74)) and 3.0-3.5 years for women (0.73 (0.72–0.75)) and decreased with increasing longitudinal windows thereafter.

**Conclusion:**

Utilising temporal changes in the commonly performed FBC test could enhance risk stratification for colorectal cancer in primary care. Further research may highlight approaches for improving predictive performance further.

**Supplementary Information:**

The online version contains supplementary material available at 10.1186/s12885-026-16179-9.

## Background

Colorectal cancer is the fourth most common type of cancer [1] and second most common cause of cancer-related death [2] in the UK. Survival is associated with tumour stage at diagnosis: five-year survival is 90% at Stage I and 10% at Stage IV [3]. Around 55% of staged cases are diagnosed late-stage, when survival is poorest [4]. Identification at earlier stages would improve likelihood of successful treatment and reduce mortality [5]. Most colorectal cancer diagnoses (55%) are made following urgent general practitioner (GP) referral for symptoms, recommended by the National Institute for Health and Care Excellence (NICE) [6]. As many symptoms of colorectal cancer are common and non-specific, many more referrals are made than cancers diagnosed, placing significant pressure on limited healthcare resources, particularly on colonoscopy capacity.

The full blood count (FBC) is a blood test measuring up to 20 components of the blood and is commonly performed in primary care [7]. Abnormalities in many individual blood components, such as low haemoglobin (anaemia) and raised platelet levels (thrombocytosis), are associated with increased risk of colorectal cancer [8]. We previously reported temporal changes (or trends) over repeat FBCs over a 10-year period for each component separately, with a statistically significant association between most trends and colorectal cancer diagnosis, with cases having on average a different trend within four years prior to diagnosis compared to patients without a diagnosis [9]. Our study suggested that relevant trends may appear before abnormal FBC thresholds [10–12] that prompt further cancer investigation [13–15] are reached, showing potential for earlier diagnosis.

We subsequently developed the BLOODTRACC models, sex-stratified diagnostic prediction models derived using joint model of longitudinal and time-to-event data and English Clinical Practice Research Datalink (CPRD) GOLD primary care database [16]. Each model utilises age and trends in three FBC components (haemoglobin, mean corpuscular volume (MCV), and platelets) to determine two-year risk of colorectal cancer. Internal validation showed that the c-statistic was 0.76 for females and 0.75 for males, using data measured earlier than two years before diagnosis. We aimed to conduct an external validation of the BLOODTRACC models to adequately assess how well they perform in external primary care datasets.

## Methods

Study reporting follows the TRIPOD guidelines [17]. Data preparation and analysis was performed in RStudio (R V4.1.3).

### Study population

Patient data was from the English CPRD AURUM primary care electronic health record database. The CPRD data included patients registered between 1st January 2000 and 31st December 2018. The data was linked to four databases: (1) National Cancer Registration and Analysis Service (NCRAS) Cancer Registration; (2) Office for National Statistics (ONS) Death Registration; (3) Hospital Episode Statistics (HES) Admitted Patient Care, Outpatient, and Accident and Emergency; (4) Small Area Level Data. SNOMED-CT, Medcodes, and ICD10 codes that were used to extract each data item are available at https://github.com/PradeepVirdee/BLOODTRACC_ModelValidation_CPRDAurum [18].

Patients were aged at least 40 years with at least one haemoglobin, MCV, and platelet measurement available in their primary care record. Patients registered with their primary care practice for less than one year, with a history of colorectal cancer before their current test (the index test), or not eligible for linkage to NCRAS, HES, and ONS were excluded.

GP practices in CPRD GOLD (derivation study) use the Vision GP system, whereas GP practices in CPRD AURUM (validation study) use the EMIS GP system. In this study, GP practices that migrated from Vision to EMIS software were excluded by the CPRD prior to transfer of the dataset. This validation study therefore offers an assessment of generalisability of the prediction models between GP systems, with reduced overlap of patients.

### Study design

We used the same design as the model derivation study. We performed a cohort study, but included an element of case-control design: we first identified the date of diagnosis in cancer cases and study exit date in cancer-free patients and excluded haemoglobin, MCV, and platelet measurements within the two years prior, ensuring we used data measured at a sufficiently earlier phase that increases the likelihood of successful clinical intervention to improve prognosis. This two-year exclusion period also reduces bias resulting from cancer patients having more tests performed more frequently in the run up to diagnosis than cancer-free patients. From the resulting dataset, the cohort was selected: the current test was the current/most recent test available. Tests performed before current test were considered historic. Trends were identified using all historical tests available up to five years prior to the current test. Risk predictions are therefore made from the current/index time-point, incorporating information from historical tests. A five-year longitudinal period was chosen based on our previous work showing differences in trends between patients with and without a diagnosis confined to five years pre-diagnosis [9]. A graphical depiction of our study design has previously been reported [16].

### Outcome

Due to the two-year exclusion window described above, there were no diagnoses or censored patients within the two years following the current test (all diagnoses and censoring occurred around the two-year mark, as described next, giving all patients in the cohort around two years of follow-up from their current test to outcomes). Therefore, the outcome was a diagnosis of colorectal cancer at two years (+/- three months to allow for a time-to-event distribution to form) after the current test. Patients without a diagnosis at two years (+/- three months) post-current were censored at the earliest of date of leaving the practice, death, 31st December 2018, or two years after their current test. Diagnoses were identified from the NCRAS database using ICD10 codes C18-C20.

### Covariates

The models rely on age (years) at the current test, sex, and trends in historic haemoglobin, MCV, and platelet measurements up to the current test. Date of birth and sex were provided for all patients by the CPRD. We cleaned blood test results to remove values outside biologically plausible ranges, which have previously been reported [9], and standardised to the same unit of measurement following guidance from previous work [19]. Missing values for these components were derived from other available components in that FBC test using established mathematical relationships between blood test components [19]. There was little ( < ~ 5%) missing haemoglobin, MCV, and platelet data among all FBCs in the resulting data so no data imputation was performed. Instead, the derived models employed joint modelling, which includes a mixed-effects modelling component that can account for sporadically measured, unbalanced, and correlated data, so every blood test result, as available, was included in this validation study. The coefficients from the derived joint models, including from the mixed-effects modelling component, were executed on the data in this study to derive risk predictions that accounted for sporadic and irregular blood testing between patients.

### Model performance

Risk predictions were derived using the *dynSurv()* command in the *JoineRML* package. This command first identifies the patient-specific trends by executing the mixed-effect sub-model coefficients on the repeat blood test data. Then, the random effects, which hold information about the trend, are extracted from the mixed-effect sub-model and entered into the linked Cox sub-model, where the Cox model coefficients (and baseline survival) are executed to use the trend and other covariates to derive event-free probabilities. These probabilities are then converted to risk of colorectal cancer by subtracting them from 1.

Overall performance was assessed using the Brier score (= 0 indicates no difference between observed and predicted risks; lower is better) [20]. Discrimination was assessed using the c-statistic (or area under the curve) (conventional rule-of-thumb of ≥ 0.7 indicates good discrimination; higher is better) and Royston and Sauerbrei’s D-statistic (higher is better) [21]. Calibration was assessed using the calibration slope (= 1 indicates perfect calibration, < 1 indicates overfitting, > 1 indicates underfitting) and calibration plots. Calibration plots were derived by first categorising patients into 20 equally sized groups of predicted two-year risk and the mean predicted two-year risk compared with the observed two-year risk for each risk group separately, with an overlaid LOWESS smoother. The observed two-year risk for each group was estimated using the Kaplan-Meier survival function to account for censored observations.

The c-statistic and calibration plots were assessed overall and in subgroups of patient data: age (40–50, 50–60, …, 90 + years), co-occurring symptoms (in the three months before the current test), and time span of repeat FBCs (6-monthly time bands from zero to five years). We assessed the c-statistic and calibration by the number of repeat tests used to define a patient-level trend (from two to 10), but as older patients are likely to have more repeat FBCs than younger patients, this analysis was stratified by age group. We compared the c-statistic of the models with that of blood test abnormality (yes/no) on the most recent test in the longitudinal window: low haemoglobin if < 13 g/dL for men and < 11.5 g/dL for women, low MCV if < 76fL, and raised platelets if > 400 × 10^9^/L [22]. The c-statistic for blood test abnormality was derived using a Cox model that included all three tests as binary variables (abnormal = yes/no), with adjustment for age at the test date. We further derived the c-statistic for early-stage (stage I-II) and late-stage (stage III-IV) cancer diagnoses.

Patients were categorised into low- vs. high- risk groups using thresholds corresponding to the 1st percentile, every 5th percentile from 5th to 95th, and 99th percentile of predicted risks. Sensitivity, specificity, positive predictive value (PPV), and negative predictive value (NPV) were derived at each threshold. Net benefit plots were also derived.

We also derived the two-year cancer incidence using patient data up to 2014 to offer a direct comparison with the cancer incidence in the derivation study, which used data over 2000–2014.

### Sensitivity analysis

We performed two sensitivity analyses. The first incorporates cancer diagnoses from CPRD, HES, and ONS, in addition to NCRAS (further details are in Supplementary Methods). In the second sensitivity analysis, we used a different study design. For the main analysis, we performed a cohort study that excluded haemoglobin, MCV, and platelet measurements within the two years prior to diagnosis in cases and study exit in cancer-free patients. This approach does not entirely reflect patient flow in practice, as patients can be diagnosed within two years of their FBC test. This sensitivity analysis therefore used a traditional cohort study design. For each patient, we identified the current/latest FBC, which was considered the index test, and trends were identified using all historical tests available up to five years prior to the current/index test. This sensitivity analysis therefore did not include a two-year washout period. The outcome was a diagnosis of colorectal cancer within two years following the current test. Patients without a diagnosis in this two-year period were censored at the earliest of date of leaving the practice, death, 31st December 2018, or two years after their current test. All other study details remained the same. A graphical depiction of the study design is in Supplementary file 1 Figure S1).

## Results

### Summary of patient data

**Fig. 1:**
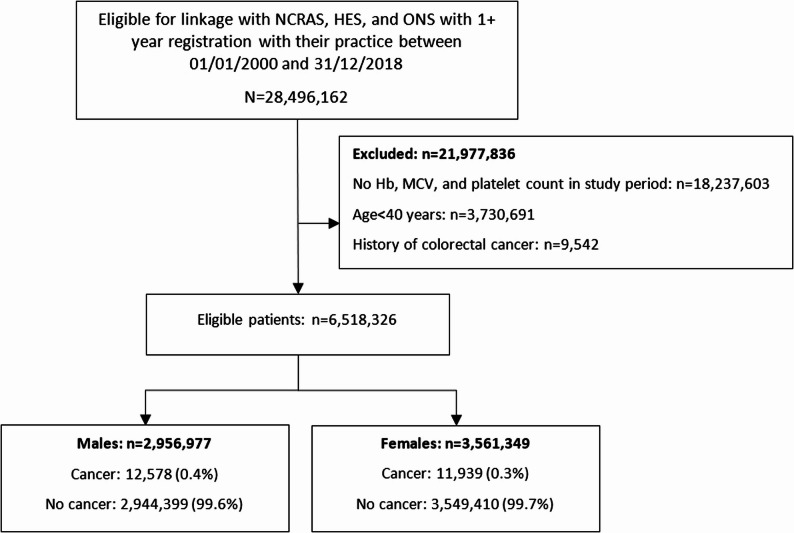
Patient flow diagram. Abbreviations: NCRAS=National Cancer Registration and Analysis Service; HES=Hospital Episode Statistics; ONS=Office of National Statistics; Hb=haemoglobin; MCV=mean corpuscular volume

We included 2,956,977 males and 3,561,349 females (Fig. 1), with 12,578 (0.4%) and 11,939 (0.3%) diagnosed with colorectal cancer at two years after their current FBC test, respectively. This event rate was comparable when restricting patient data to 2000–2014: 0.5% males and 0.4% females. Patients with colorectal cancer were on average around 10 years older than patients without (Table 1). Median time (years) from first to last blood test used to derive trends was higher in cases than cancer-free patients: male cases 3.2 and cancer-free 2.7; female cases 3.0 and cancer-free 2.1 (Supplementary file 1 Table S1).

**Table 1:** Summary of patient data

	Males (*n*=2,956,977)		Females (*n*=3,561,349)	
	Diagnosed	Not diagnosed	Diagnosed	Not diagnosed
Total patients	12,578 (0.4%)	2,944,399 (99.6%)	11,939 (0.3%)	3,549,410 (99.7%)
Age (years) at current FBC				
Mean (SD)	71.6 (10.3)	61.6 (13.7)	73.9 (11.3)	62.7 (15.0)
Median (IQR)	72 (65-79)	60 (50-72)	76 (87-82)	61 (50-75)
Ethnicity, n (%)				
Asian	78 (0.6%)	46,355 (1.6%)	75 (0.6%)	52,873 (1.5%)
Black	203 (1.6%)	95,943 (3.3%)	201 (1.7%)	119,413 (3.4%)
Mixed	28 (0.2%)	17,342 (0.6%)	31 (0.3%)	22,373 (0.6%)
Other	81 (0.6%)	44,823 (1.5%)	77 (0.6%)	50,244 (1.2%)
South Asian	218 (1.7%)	106,851 (3.6%)	151 (1.3%)	117,228 (3.3%)
White	11,691 (93.0%)	2,407,565 (81.8%)	10,961 (91.8%)	2,907,510 (81.9%)
Unknown	279 (2.2%)	225,520 (7.7%)	443 (3.7%)	279,769 (7.9%)
IMD quintile				
1 (richest)	2,835 (21.1%)	641,060 (21.8%)	2,776 (21.5%)	783,492 (22.1%)
2	2,833 (21.1%)	628,975 (21.4%)	2,719 (21.1%)	763,896 (21.5%)
3	2,736 (20.4%)	578,092 (19.6%)	2,595 (20.1%)	697,553 (19.7%)
4	2,565 (19.1%)	565,080 (19.2%)	2,459 (19.0%)	678,795 (19.1%)
5 (poorest)	2,429 (18.1%)	525,951 (17.9%)	2,347 (18.2%)	619,595 (17.5%)
Unknown	25 (0.2%)	4,396 (0.2%)	23 (0.2%)	5,099 (0.1%)
Symptom, n (%)				
Any CRC symptom	782 (6.6%)	203,777 (6.9%)	972 (8.3%)	269,223 (7.6%)
Abdominal pain	273 (2.2%)	88,848 (3.0%)	391 (3.2%)	134,010 (3.8%)
Appetite loss	26 (0.2%)	3,737 (0.1%)	20 (0.2%)	5,597 (0.2%)
Change in bowel habit	62 (0.5%)	14,162 (0.5%)	56 (0.5%)	16,581 (0.5%)
Constipation	152 (1.2%)	26,515 (0.9%)	168 (1.4%)	37,024 (1.0%)
Diarrhoea	136 (1.1%)	35,511 (1.2%)	212 (1.8%)	48,016 (1.4%)
Rectal bleeding	126 (1.0%)	26,005 (0.9%)	137 (1.2%)	21,859 (0.6%)
Weight loss	65 (0.5%)	19,423 (0.7%)	74 (0.6%)	21,164 (0.6%)
Cancer stage, n (%)				
I	1,732 (13.8%)		1,348 (11.3%)	
II	2,904 (23.1%)		2,651 (22.2%)	
III	2,968 (23.6%)		2,787 (23.3%)	
IV	2,204 (17.5%)		1,999 (16.7%)	
Unknown	3,615 (22.0%)		4,134 (26.4%)	

Abbreviations: *SD* standard deviation, *IQR* interquartile range, *CRC* colorectal cancer

### Model performance

**Fig. 2:**
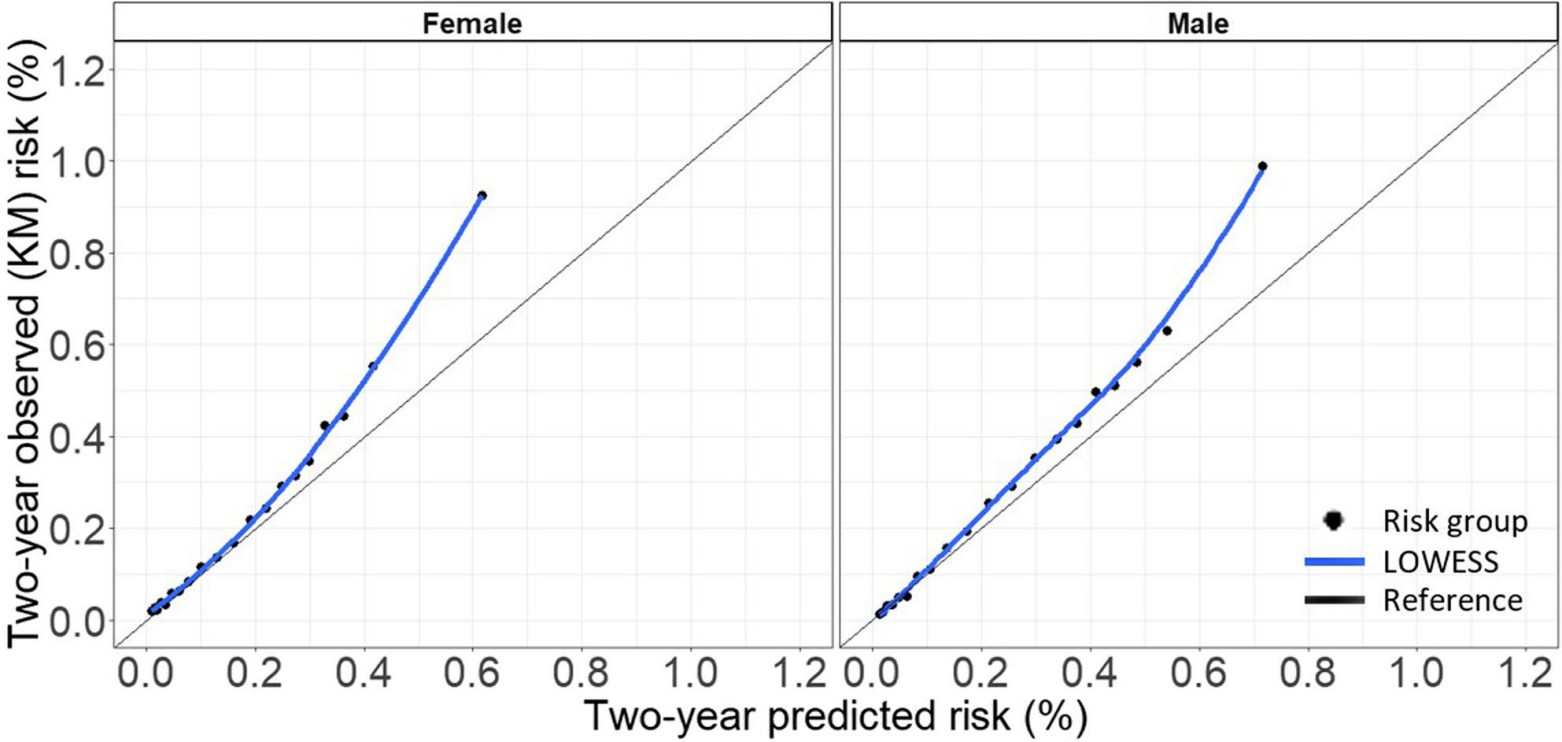
Calibration plots for the BLOODTRACC models. Abbreviations: KM=Kaplan-Meier

The median (range) of predicted risk for men was 0.4% (0.1–5.7) among cases and 0.2% (0.1–7.7) among cancer-free patients. For women, this was 0.3% (0.1–4.9) among cases and 0.1% (0.1–9.9) among cancer-free patients. The c-statistic (95% CI) was 0.73 (0.72–0.73) for males and 0.74 (0.74–0.75) for females (Table 2). The calibration slope (95% CI) was 0.92 (0.89–0.94) for males and 0.95 (0.93–0.98) for females. Calibration plots indicated that under-prediction increased as the risk group increased and was largest for the highest risk group (Fig. 2): 0.14% predicted vs. 0.16% observed risk in males and 0.10% predicted vs. 0.12% observed risk in females. In males, the c-statistic (95% CI) was 0.72 (0.72–0.73) for early-stage diagnoses and 0.71 (0.71–0.72) for late-stage. In females, it was 0.74 (0.73–0.74) for early-stage diagnoses and 0.72 (0.71–0.72) for late-stage.

**Table 2:** Performance measures (95% CI) of the BLOODTRACC models

Performance measure	Males	Females
Brier score	0.0042	0.0033
C-statistic	0.73 (0.72 - 0.73)	0.74 (0.74 - 0.75)
D-statistic	1.18 (1.16 - 1.21)	1.32 (1.29 - 1.35)
Calibration slope	0.92 (0.89-0.94)	0.95 (0.93-0.98)

### Model performance in patient subgroups

The c-statistic generally decreased as males and females grew older, ranging 0.54–0.63 and 0.58–0.63, respectively (Supplementary file 1 Figure S2). Calibration plots showed good calibration in patients aged < 50 years, with increasing under-prediction with increasing age, with most under-prediction observed for patients aged 80–89 years and greater for men than women (Supplementary file 1 Figure S3). The c-statistic by ethnic group, IMD quintile, symptom presence, and number of blood tests available used to derive the trend as in Supplementary file 1 Figures S4-S8.

When we assessed the c-statistic by the time span encompassing the repeat tests, the c-statistic was similar for all time spans up to 2.0-2.5 years (range 0.74–0.76) for men and 2.5-3.0 years (range 0.72–0.77) for women, but decreased as the time span of repeat tests grew larger thereafter: 0.73 (95% CI = 0.71–0.74) for repeat tests over 2.5-3.0 years to 0.68 (95% CI = 0.67–0.69) over 4.5-5.0 years in men and 0.73 (95% CI = 0.72–0.75) for repeat tests over 3.0-3.5 years to 0.70 (95% CI = 0.69–0.71) over 4.5-5.0 years in women (Supplementary file 1 Figure S9). The models were well calibrated among patients with up to three repeat tests, with increasing under-prediction as the number of repeat tests increased, and among patients with all their repeat tests confined within a six-month period, with under-prediction observed for longer repeat testing periods (Supplementary file 1 Figure S10).

### Comparison to abnormal test results

For each age group, the c-statistic was comparable between the BLOODTRACC models, which included blood test trends (adjusted for age and sex), and Cox models, which considered the abnormality counterparts (Supplementary file 1 Figure S8). However, the c-statistic for the BLOODTRACC models appeared to become greater than for Cox models including abnormality in women aged at least 70 years.

### Diagnostic accuracy

The highest risk percentile, 99%, corresponded to a risk threshold of 0.7810% for males and 0.6872% for females, with 3.56% and 5.03% sensitivity and 99.01% and 99.01% specificity, respectively (Supplementary file 1 Table S2 and Table S3). The PPV increased as the threshold used to define low- vs. high-risk increased, ranging 0.43–1.52% for men and 0.34–1.68% for women. The NPV ranged 99.59–99.98% for men and 99.68–99.96% for women, indicating a high proportion of patients with low predicted risk without an observed diagnosis regardless of the threshold used to define low- vs. high-risk, likely due to the low event rate overall. Net benefit curves indicate that the models would be clinically useful, offering greater benefit than the default “investigate all” and “investigate no one” strategies (Figure S11).

### Sensitivity analysis

Including cancer diagnoses from NCRAS, CPRD, HES, and ONS resulted in the inclusion of 2,956,977 men and 3,561,349 women, with the number with a cancer diagnosis increasing from 12,578 (0.4%) and 11,939 (0.3%) to 13,423 (0.5%) and 12,919 (0.4%), respectively (Supplementary file 1 Table S4). The c-statistic (95% CI) increased slightly to 0.747 (0.744–0.750) for males and remained similar at 0.740 (0.736–0.744) for females. The calibration slope (95% CI) was 0.91 (0.88–0.93) for males and 0.94 (0.92–0.97) for females. Calibration plots indicated that the models consistently under-predicted risk, with under-prediction worsening from the main analysis, as expected due to the inclusion of further observed diagnoses. Under-prediction was greatest for the highest risk group (Supplementary file 1 Figure S12): 0.69% predicted vs. 1.28% observed risk in males and 0.62% predicted vs. 1.00% observed risk in females.

The second sensitivity analysis using a cohort study design included 5,011,297 men and 7,010,202 women, of which 0.4% (*n* = 18,876) and 0.3% (*n* = 17,501) were diagnosed with colorectal cancer within two years following their current/index blood test, respectively (Supplementary file 1 Table S5). This was comparable to the main analysis using a hybrid case-control and cohort study design: 0.4% in men and 0.3% in women. This approach, which included data measured closer to diagnosis, increased the c-statistic (95% CI) from 0.73 (0.72–0.73) to 0.84 (0.83–0.85) in men and from 0.74 (0.74–0.75) to 0.91 (0.89–0.93) in women.

## Discussion

### Summary of main findings

Our prediction models have good discriminative ability for two-year risk of colorectal cancer, based on only age, sex, and trends in haemoglobin, MCV, and platelet count earlier than two years before diagnosis/censor, and performed slightly better for early-stage diagnoses than late-stage. The models however under-predicted two-year risk in the patients with higher predicted risks, likely reflecting older patients, as calibration in younger patients was good. The two-year event rate remained the same when considering only patient data over 2000–2014, coinciding with the prior derivation study period, and was comparable to the derivation study (0.4% in men, 0.3% in women) [16]. This under-prediction may be explained by practice-level differences between practices contributing to the CPRD GOLD and AURUM databases. Our net benefit curves indicate that the models would be clinically useful, offering greater benefit than the default “treat all” and “treat no one” strategies. However, unlike the NICE-recommended 3% risk referral threshold for gastrointestinal cancers combined in patients with symptoms [6], no recommended threshold has been established for colorectal cancer specifically in a blood tested cohort.

Discrimination of the models generally decreased within older patient subgroups, with older patients being likelier to have other (unaccounted) conditions that could influence FBC test results, making it more challenging to capture a cancer-related trend compared to younger patients. Discrimination was comparable by IMD quintile and for most ethnic groups, but lowest for South Asian women, possibly as South Asian populations have a higher prevalence of low haemoglobin and low MCV, making a declining haemoglobin and MCV trend less predictive of colorectal cancer [23]. Among symptom groups, discrimination was highest for those with co-occurring rectal bleeding, as expected, as this is an established ‘red flag’ or ‘alarm’ symptom for colorectal cancer [6]. Discrimination was slightly poorer for patients with non-specific symptoms, such as appetite loss, constipation, and diarrhoea, as these symptoms could be related to many other conditions. Ongoing work is investigating the role of blood test trend to improve cancer risk stratification in patients with non-specific symptoms [24].

### Strengths and limitations

A key strength of this study was the large sample size and follow-up duration used to validate the prediction models. This allowed us to explore the impact of patient age and varying time periods and number of tests capturing a trend on model performance. One limitation is that the reason for the FBC being ordered in primary care is unknown. The FBC is a non-specific test, so are ordered in primary care for many reasons and not specifically for colorectal cancer. Knowing the reason for testing could help identify other conditions that could influence blood test trends. Additionally, it is possible that patients without colorectal cancer who have many FBCs in the five-year period have another disease or condition that influences blood levels over time. Therefore, some false positives (patients determined to be high risk who are not diagnosed with colorectal cancer) may have another illness. Data on comorbidities, including other cancers, will be accounted for as future work. A further limitation is that we were unable to assess how the models perform alongside faecal immunochemical testing (FIT) testing. Our dataset ends in 2018, around the time that FIT testing was implemented, including to rule out colorectal cancer in those with low-risk symptoms. We therefore did not have enough FIT test data to make this analysis achievable.

### Comparison with existing literature

Our systematic review identified 13 prediction models that use some FBC data to inform risk of colorectal cancer [25]. All of these models are static, using a single test from one time point per patient, except the ColonFlag model, a machine-learning algorithm, derived using data from an Israeli population, designed to predict three-to-six month colorectal cancer risk based on changes in all FBC components measured at 36 and 18 months before a patient’s current FBC [26]. The use of repeated measures data can provide more individualised risk predictions, so our models may offer improved risk stratification to existing static models (using covariates measured at one time point). However, predictive performance of our dynamic BLOODTRACC models is yet to be compared to that of static models in the same patient cohort using similar study designs to reduce heterogeneity.

We previously reported an external validation of the ColonFlag, performed in the same split sample internal validation cohort used in the BLOODTRACC model derivation study to offer a direct comparison of model performance [16]. The c-statistic for two-year risk was comparable between our models and the ColonFlag. In men, it was 0.75 for BLOODTRAC in this external validation, 0.75 for BLOODTRACC in its derivation study, and 0.76 for ColonFlag in its previous external validation. For women, it was 0.74 for BLOODTRAC in this external validation, 0.76 for BLOODTRACC in its derivation study, and 0.76 for ColonFlag in its previous external validation. Performance of each model was also similar in subgroups of age, number of FBCs used to derive trend, and length of the longitudinal window. Comparable performance was expected, as the models use the same data (age, sex, changes over time in FBC tests) to identify risk, although the ColonFlag includes trends in all 20 FBC parameters to identify risk [26] and our models are simpler, using only haemoglobin, MCV, and platelets. Discrimination remained comparable regardless, suggesting these additional FBC parameters may not enhance risk estimation. The ColonFlag model is commercially developed and not publicly available. The BLOODTRACC models may increase the likelihood of adoption and embedding within electronic health record systems, facilitate flagging of cancer risk in practice.

Our second systematic review, which focused on clinical prediction models incorporating trends over repeated blood tests (liver function, renal function, and FBC) to inform cancer diagnosis, did not identify any additional existing trend-based prediction models for colorectal cancer, other than our current BLOODTRACC models [16] and the ColonFlag algorithm [26].

### Implications for practice

Our dynamic prediction models are designed to provide an up-to-date risk prediction each time a new haemoglobin, MCV, or platelet measurement is added to a patient’s GP record. Using combinations of trends over repeated tests could identify subtle simultaneous cancer-relevant changes in tests that could otherwise be missed in practice, including changes within the normal reference range, which are unlikely to be noticed by a clinician. The models are designed to be pragmatic and use routinely available data readily available in primary care, accounting for the sporadic and irregular nature of blood testing in primary care, and we plan for them to be programmed into practice software to run automatically when a new FBC becomes available. Therefore, there will likely be minimal additional work for patients or GP staff to identify a patient’s risk of undiagnosed colorectal cancer.

FIT testing, which examines stool samples for traces of blood, has proved a useful test outside the screening programme for ruling out colorectal cancer in patients with symptoms attending primary care, based on a 98% NPV in those with a negative FIT in a recent primary care study [27, 28]. Patients identified as high-risk from our models could be offered a FIT test, which is much more practical, cheaper and less invasive than colonoscopy. As the use of FIT increases in clinical practice and FIT results are recorded in the primary care electronic health record, research should investigate the additional diagnostic value of adding historical blood test trend to the FIT value at the time of clinical presentation. This would complement past and ongoing efforts to increase the predictive value of FIT by combining it in a model with patient characteristics and blood tests taken at the time of the FIT [29–32]. Further work could also aim to modify the FIT threshold considered ‘positive’ in high-risk patients identified from the BLOODTRACC models. Future work should compare net benefit between FIT testing and the models and assessment of net benefit in combination.

Prediction models should be updated regularly to incorporate changes in clinical practice [33]. The models have good discriminative ability using only data measured earlier than two years before diagnosis, but discrimination could be improved by updating the model to include data closer to diagnosis, enhancing risk stratification. For example, our sensitivity analysis using a traditional cohort study design in essence extended the trend to capture changes measured closer to diagnosis and allows for shorter-term cancer diagnoses, which improved the c-statistic. This approach would introduce bias in that cancer cases have more frequent testing than cancer-free patients in routinely collected data. However, the updated models could include the number of repeat tests used to derive each patient’s trend as a covariate to adjust for this imbalance. As model discrimination was slightly better for younger individuals, BLOODTRAC may have an important role in enhancing the detection of early-onset colorectal cancer, which is rising in incidence [34]. Accounting for comorbidity in the model may improve discrimination in older patients, where blood test abnormalities are less likely to be caused by cancer than in younger less comorbid patients. These updates could also reduce the degree of under-prediction identified in this study.

## Conclusion

We externally validated dynamic clinical risk prediction models designed to inform referral in primary care. The models performed well, relying only on data measured earlier than two years before diagnosis, but did not outperform blood test abnormality, recommended in referral guidelines to inform referral. Extending the trend closer to diagnosis may enhance predictive performance and reduce under-prediction.

## Supplementary Information

Supplementary Material 1: Figure S1. Study design differences between the main and sensitivity analysis; Figure S2: C-statistic for the BLOODTRACC models by age (years) at the current test in males (top) and females (bottom); Figure S3: Calibration plots for the BLOODTRACC models by age (years) at the current test, ethnicity, and IMD quintile in males (left) and females (right); Figure S4: C-statistic for the BLOODTRACC models by ethnicity in males (top) and females (bottom); Figure S5: C-statistic for the BLOODTRACC models by IMD quintile in males (top) and females (bottom); Figure S6: C-statistic for the BLOODTRACC models by presence of co-occurring symptoms1 in males (top) and females (bottom); Figure S7: C-statistic for the BLOODTRACC models by number of FBCs per age group in males; Figure S8: C-statistic for the BLOODTRACC models by number of FBCs per age group in females; Figure S9: C-statistic for the BLOODTRACC models by time span of FBCs in males (top) and females (bottom); Figure S10: Calibration plots for the BLOODTRACC models by number of repeat tests and testing period in males (left) and females (right); Figure S11: Net benefit plots; Figure S12: Calibration plots for the BLOODTRACC models – sensitivity analysis (NCRAS+CPRD + HES+ONS cancers); Table S1: Summary of haemoglobin, MCV, and platelet data and follow-up time; Table S2: Diagnostic accuracy (95% CI) measures for males; Table S3: Diagnostic accuracy (95% CI) measures for females; Table S4: Summary of cancer diagnosis by age group; Table S5: Summary of cancer diagnosis by age group.

Supplementary Material 2. TRIPOD Checklist.

## Data Availability

The dataset used is available from the authors but is subject to access approval by the CPRD [35].

## Abbreviations

CPRD: Clinical Practice Research Datalink
CI: Confidence interval
FIT: Faecal immunochemical testing
FBC: Full blood count
GP: General practitioner
HES: Hospital Episode Statistics
Hb: Haemoglobin
Plt: Platelets
IQR: Inter-quartile range
MCV: Mean corpuscular volume
NCRAS: National Cancer Registration and Analysis Service
NICE: National Institute for Health and Care Excellence
NPV: Negative predictive value
ONS: Office for National Statistics
PPV: Positive predictive value
SD: Standard deviation
