# Supplementary material for: Full BLOOD count TRends for colorectal cAnCer deteCtion (BLOODTRACC): external validation of dynamic clinical prediction models for early detection of colorectal cancer in primary care
Source: BMC Cancer. 2026 May 14;26:774. doi: 10.1186/s12885-026-16179-9 (PMC13295383; doi:10.1186/s12885-026-16179-9)
Supplement: Supplementary file 1 — Supplementary Material 1: Figure S1. Study design differences between the main and sensitivity analysis; Figure S2: C-statistic for the BLOODTRACC models by age (years) at the current test in males (top) and females (bottom); Figure S3: Calibration plots for the BLOODTRACC models by age (years) at the current test, ethnicity, and IMD quintile in males (left) and females (right); Figure S4: C-statistic for the BLOODTRACC models by ethnicity in males (top) and females (bottom); Figure S5: C-statistic for the BLOODTRACC models by IMD quintile in males (top) and females (bottom); Figure S6: C-statistic for the BLOODTRACC models by presence of co-occurring symptoms1 in males (top) and females (bottom); Figure S7: C-statistic for the BLOODTRACC models by number of FBCs per age group in males; Figure S8: C-statistic for the BLOODTRACC models by number of FBCs per age group in females; Figure S9: C-statistic for the BLOODTRACC models by time span of FBCs in males (top) and females (bottom); Figure S10: Calibration plots for the BLOODTRACC models by number of repeat tests and testing period in males (left) and females (right); Figure S11: Net benefit plots; Figure S12: Calibration plots for the BLOODTRACC models – sensitivity analysis (NCRAS+CPRD + HES+ONS cancers); Table S1: Summary of haemoglobin, MCV, and platelet data and follow-up time; Table S2: Diagnostic accuracy (95% CI) measures for males; Table S3: Diagnostic accuracy (95% CI) measures for females; Table S4: Summary of cancer diagnosis by age group; Table S5: Summary of cancer diagnosis by age group. [file 12885_2026_16179_MOESM1_ESM.docx]

# Supplementary Methods – sensitivity analysis

*Sensitivity analysis 1:* The main analysis uses cancers from NCRAS to offer a direct comparison to the performance measures derived in the derivation study, as only cancers from the NCRAS database were used for model derivation. In our CPRD AURUM external validation dataset, cancers were also available from CPRD, HES, and ONS databases. We therefore performed a sensitivity analysis to include diagnoses from these additional sources. We first re-derived the validation cohort, excluding patients with a history of cancer from NCRAS, CPRD, HES, or ONS. The outcome was defined as before, except diagnoses were primarily selected from the NCRAS database and additional diagnoses were taken as the earliest diagnosis in CPRD, HES, or ONS if not identified in NCRAS.

# Supplementary Results – performance in subgroups

*Model performance in subgroups:* The models slightly over-predicted risk in each non-White ethnic group, but under-estimated in White patients and higher IMD quintiles. The c-statistic (95% CI) across ethnic groups ranged 0.72-0.79 for men and 0.68-0.81 for women (Supplementary file 1 Figure S4), lowest for White men (0.72 (0.72-0.72)) and South Asian women (0.68 (0.64-0.72)), and similar in IMD quintile groups (Supplementary file 1 Figure S5). The c-statistic (95% CI) was higher in men without constipation (with 0.66 (0.62-0.70); without 0.75 (0.74-0.75)) or with rectal bleeding (with 0.79 (0.76-0.83); without (0.75 (0.74-0.75)) and women without appetite loss (with 0.62 (0.52-0.73); without (0.74 (0.74-0.74)), constipation (with 0.66 (0.62-0.69); without (0.74 (0.74-0.74)), or diarrhoea (with 0.69 (0.66-0.72); without (0.74 (0.74-0.74)) (Supplementary file 1 Figure S6). The symptom subgroups that had the highest c-statistics were rectal bleeding and abdominal pain for both men and women. For each age group, the c-statistic in subgroups formed by the number of repeat tests available per patient that were used to derive trends ranged 0.56-0.72 if aged 40-49 years and 0.50-0.70 if aged 90+ years in men and 0.53-0.73 if aged 40-49 years and 0.56-0.65 if aged 90+ years in women (Supplementary file 1 Figure S7 and Figure S8).

# Figure S1: Study design differences between the main and sensitivity analysis

Patient 1

Patient 2

Patient 3

*Years*

-5

0

*(Current test)*

2

*5-year longitudinal period*

*2-year risk*

-4

-3

-2

-1

1

3

*Cancer diagnosis/censor*

Patient 1

Patient 2

Patient 3

*Years*

-6

-1

1

*5-year longitudinal period*

*2-year risk*

-5

-4

-3

-2

0

*(Current test)*

2

*Cancer diagnosis/censor*

***Main analysis***

***Sensitivity analysis***

# Table S1: Summary of haemoglobin, MCV, and platelet data and follow-up time

|  | **Validation study** | | | | **Derivation study** | | | |
| --- | --- | --- | --- | --- | --- | --- | --- | --- |
|  | **Males (n=2,956,977)** | | **Females (n=3,561,349)** | | **Males (n=250,716)** | | **Females (n=246,695)** | |
|  | **Diagnosed** | **Not diagnosed** | **Diagnosed** | **Not diagnosed** | **Diagnosed** | **Not diagnosed** | **Validation** | **Derivation** |
| **N (%) of total patients** | 12,578 (0.4%) | 2,944,399 (99.6%) | 11,939 (0.3%) | 3,549,410 (99.7%) | 865 (0.4%) | 249,851 (99.6%) | 677 (0.3%) | 246,018 (99.7%) |
| **Mean (SD) age (years) at current FBC:** | 71.6 (10.3) | 61.6 (13.7) | 73.9 (11.3) | 62.7 (15.0) | 70.9 (10.0) | 60.7 (13.0) | 73.2 (11.0) | 61.9 (14.6) |
| **No. tests in total** | 11,985 | 3,557,891 | 12,614 | 2,951,603 | 3,774 | 801,390 | 3,573 | 909,569 |
| **Mean (SD) no. tests** | 5.9 (7.2) | 4.2 (5.0) | 5.3 (6.2) | 3.7 (4.4) | 5.6 (7.8) | 4.4 (6.8) | 7.0 (9.0) | 5.3 (8.1) |
| **Median time (range)^1^** | 3.2 (0-5.0) | 2.7 (0-5.0) | 3.0 (0-5.0) | 2.1 (0-5.0) | 2.2 (0-5.0) | 1.5 (0-5.0) | 2.7 (0-5.0) | 2.0 (0-5.0) |
| **Median follow-up (range)^2^** | 5.18 (1.75-7.24) | 4.69 (1.75-7.25) | 5.00 (1.75-7.25) | 4.11 (1.75-7.25) | 4.18 (1.75-7.18) | 3.47 (1.75-7.25) | 4.67 (1.76-7.21) | 4.01 (1.75-7.25) |

^1^Time (years) between the first and last FBC in the five-year longitudinal period.

^2^Time (years) from first FBC to diagnosis/censor date.

Abbreviations: MCV=mean corpuscular volume; SD=standard deviation

Derivation study results are from: [Virdee et al. DOI: 10.3390/cancers14194779]

# Figure S2: C-statistic for the BLOODTRACC models by age (years) at the current blood test (index)


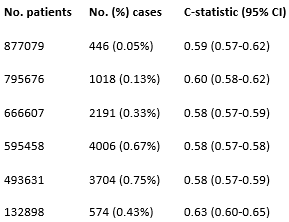

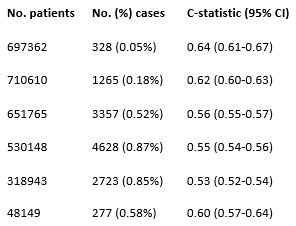

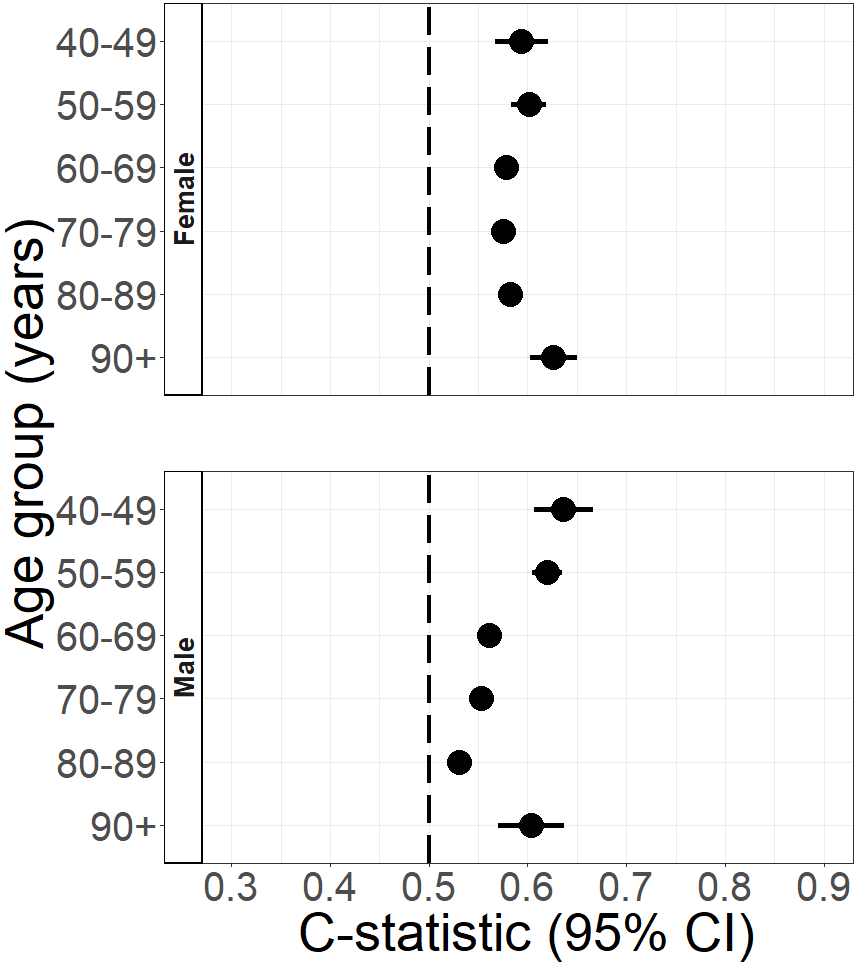


# Figure S3: Calibration plots for the BLOODTRACC models by age (years) at the current blood test, ethnicity, and IMD quintile


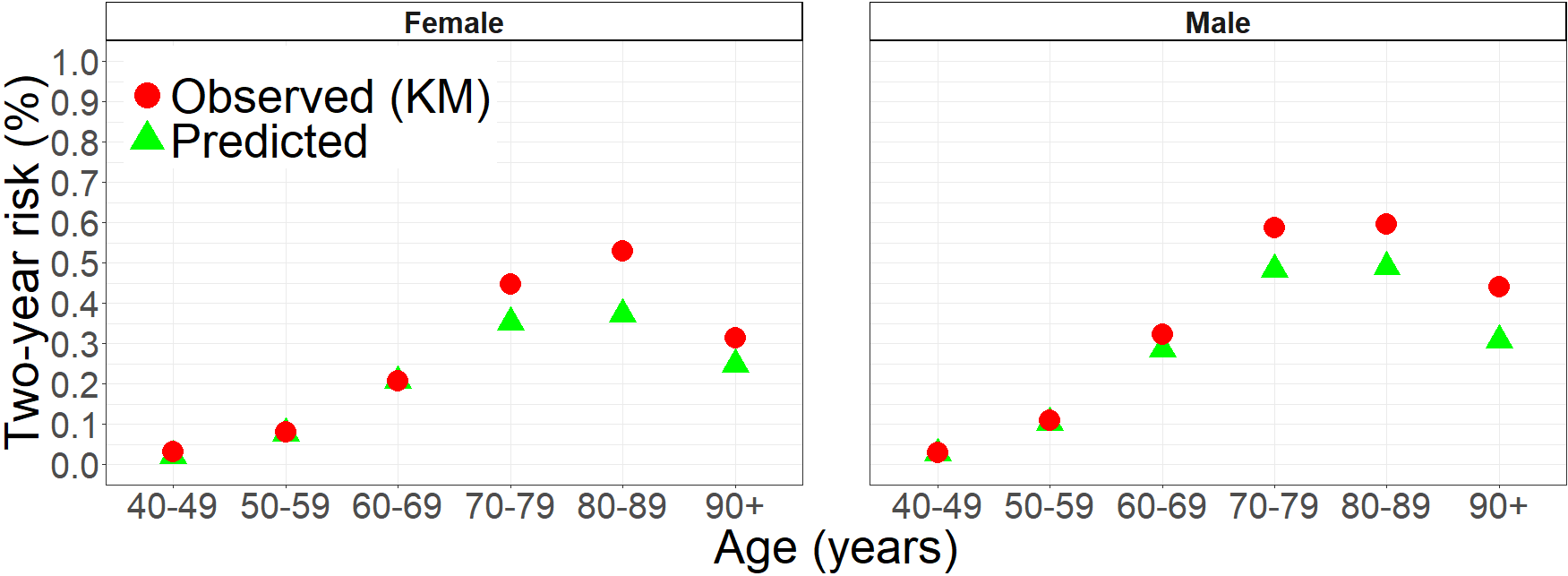

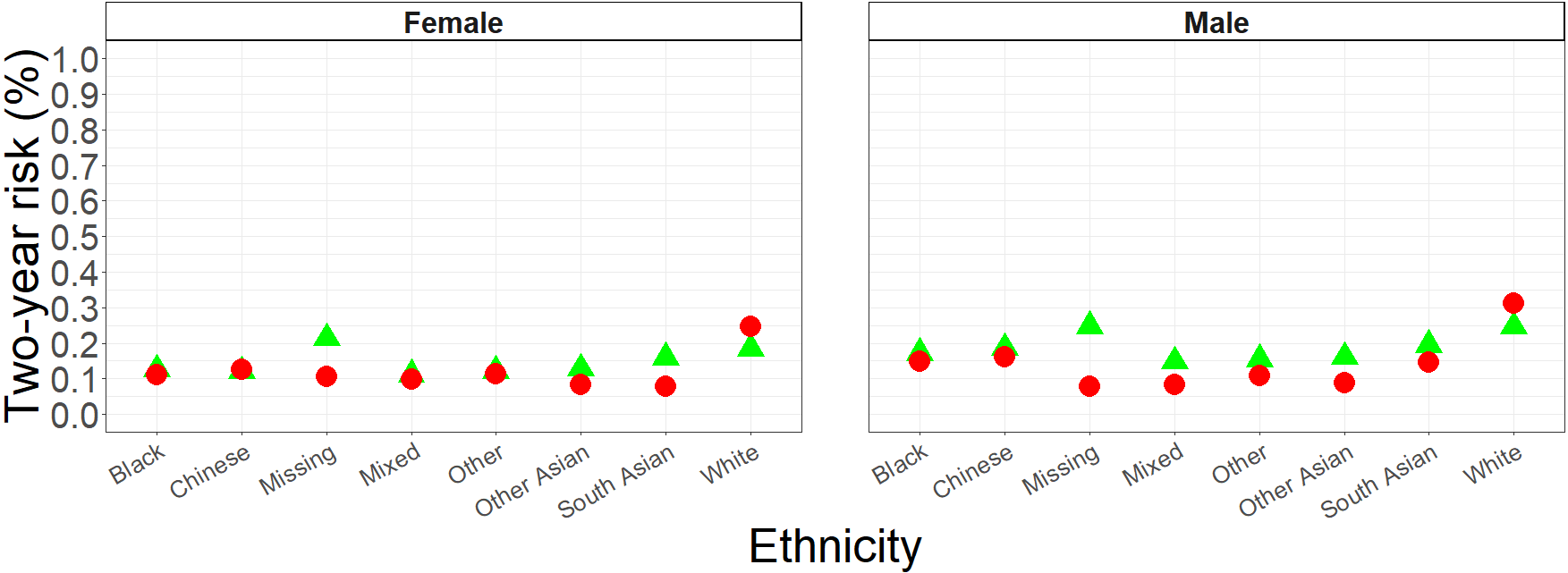

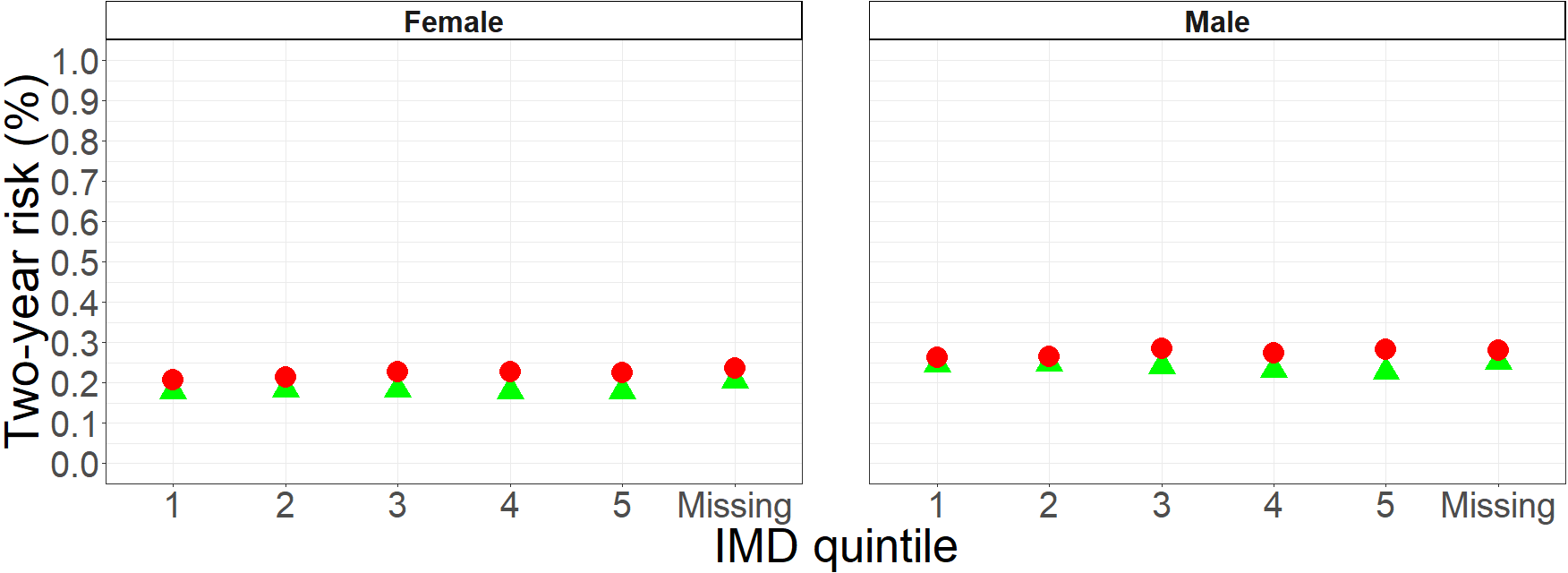


Abbreviations: KM=Kaplan-Meier

# Figure S4: C-statistic for the BLOODTRACC models by ethnicity


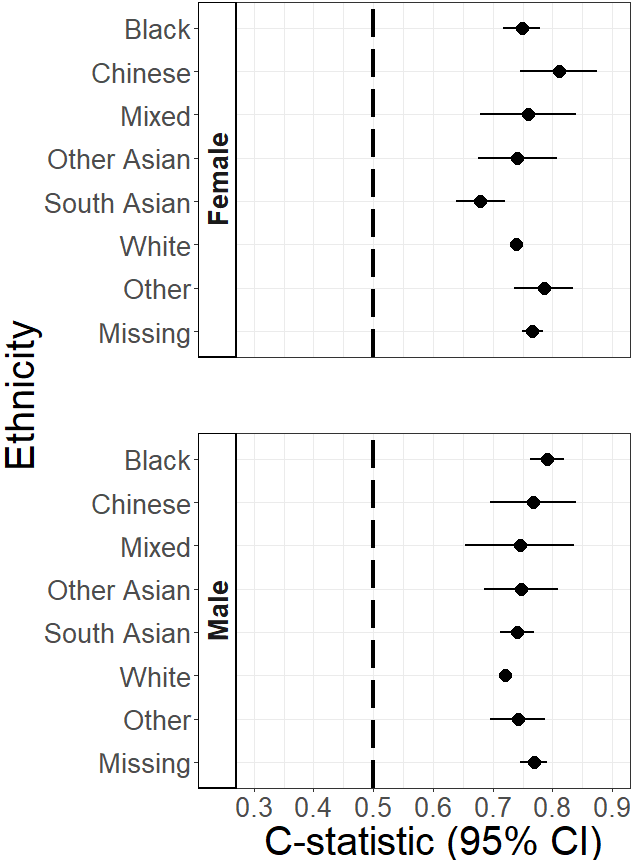

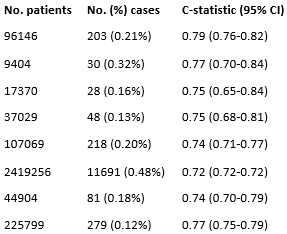

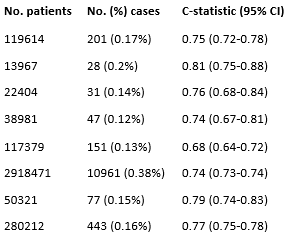


# Figure S5: C-statistic for the BLOODTRACC models by IMD quintile


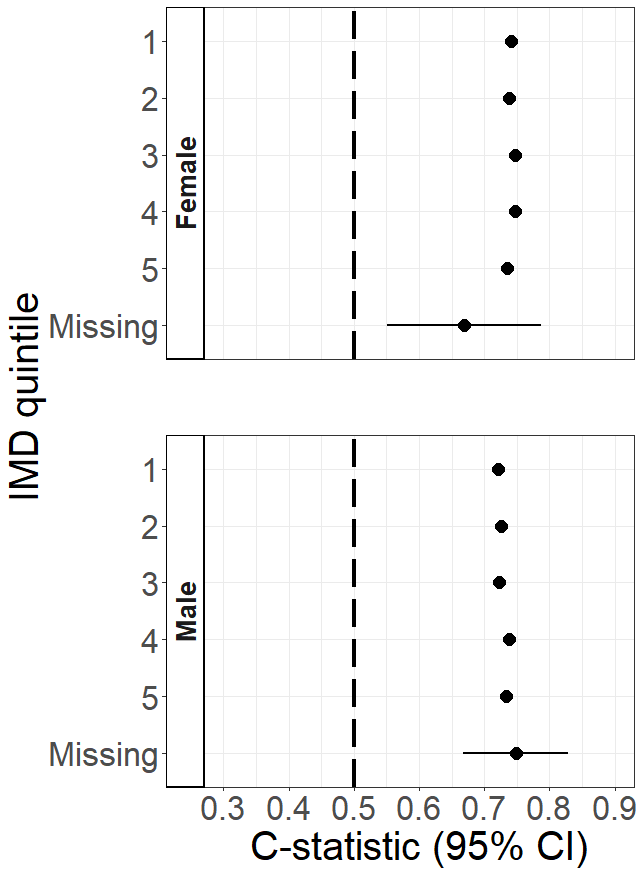

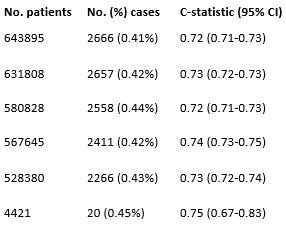

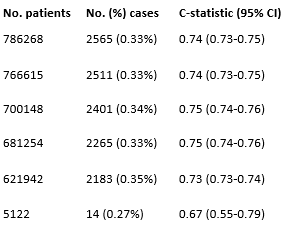


# Figure S6: C-statistic for the BLOODTRACC models by presence of co-occurring symptoms^1^


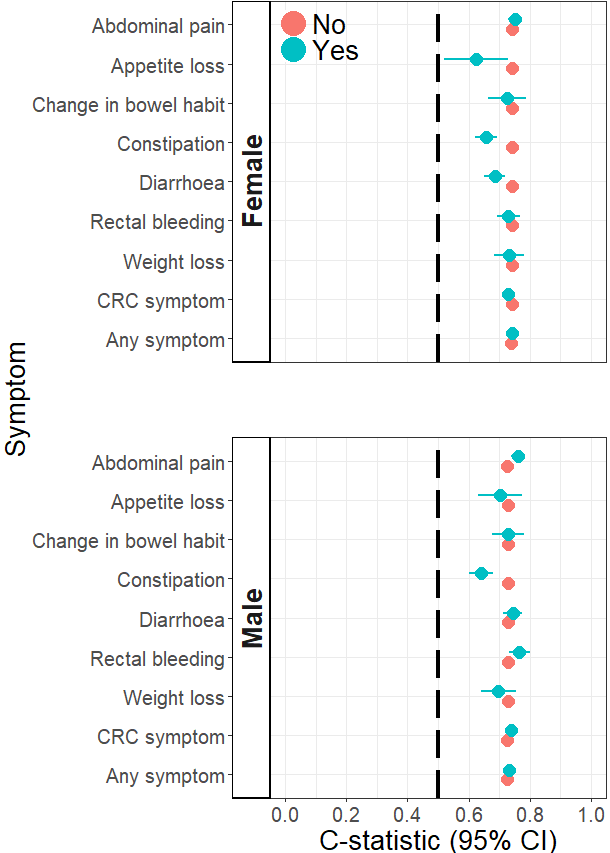

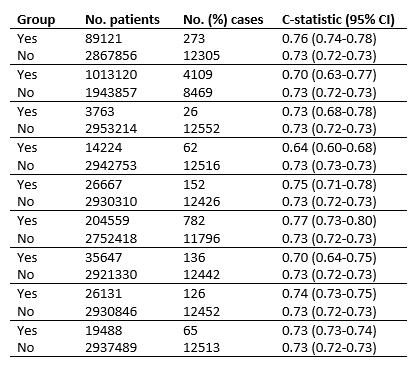

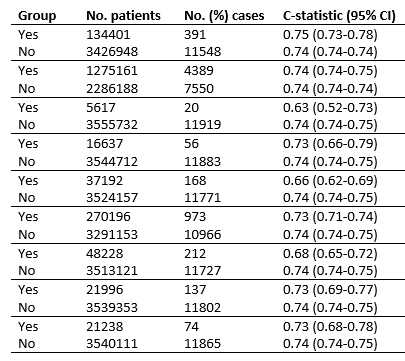


^1^Presence (=yes) or no presence (=no) of the symptom within the three months prior to the current (index) test.

# Figure S7: C-statistic for the BLOODTRACC models by number of FBCs per age group in males


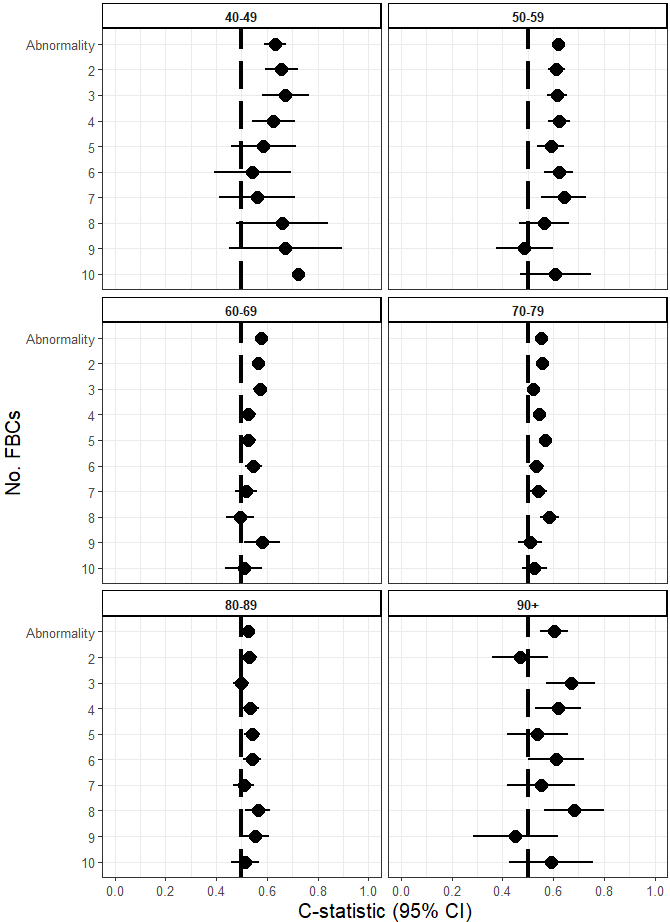


Abbreviations: FBC=full blood count.

# Figure S8: C-statistic for the BLOODTRACC models by number of FBCs per age group in females


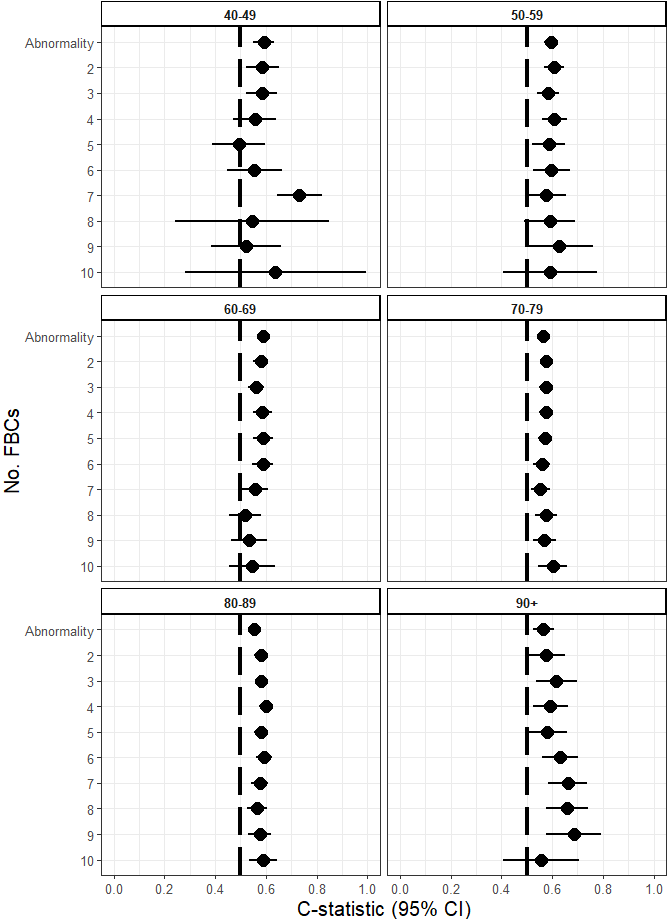


Abbreviations: FBC=full blood count.

# Figure S9: C-statistic for the BLOODTRACC models by time span of FBCs


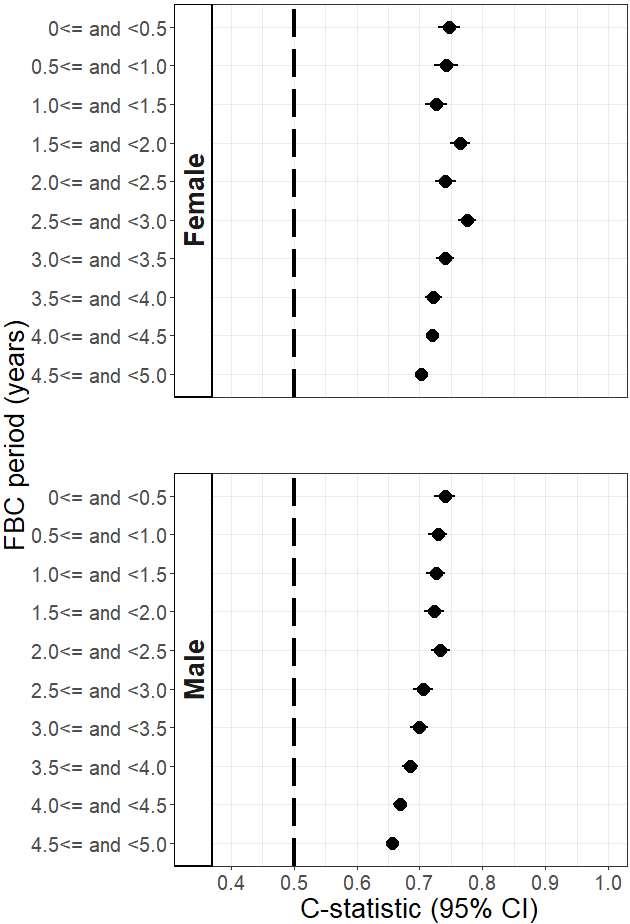

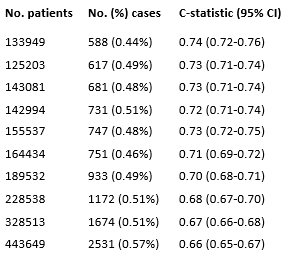

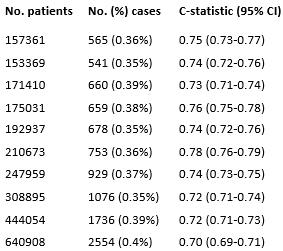


Abbreviations: FBC=full blood count.

# Figure S10: Calibration plots for the BLOODTRACC models by number of repeat tests and testing period


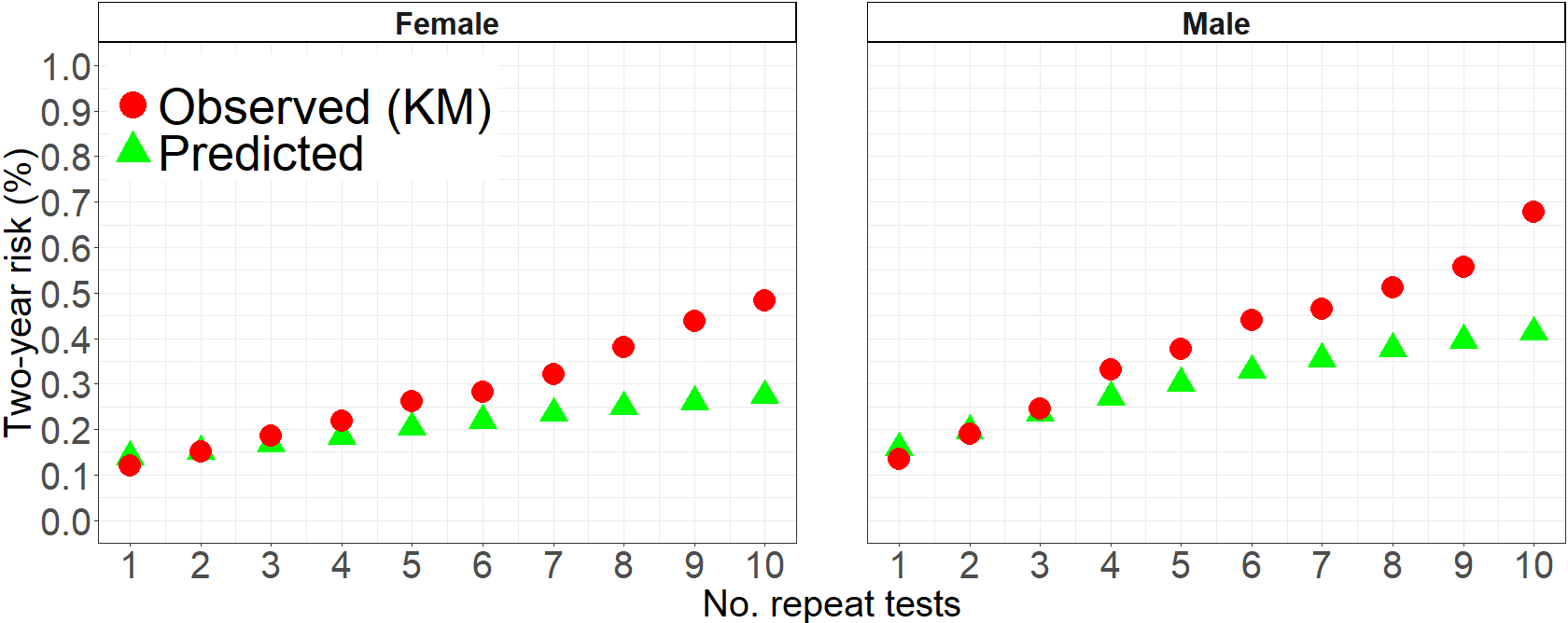

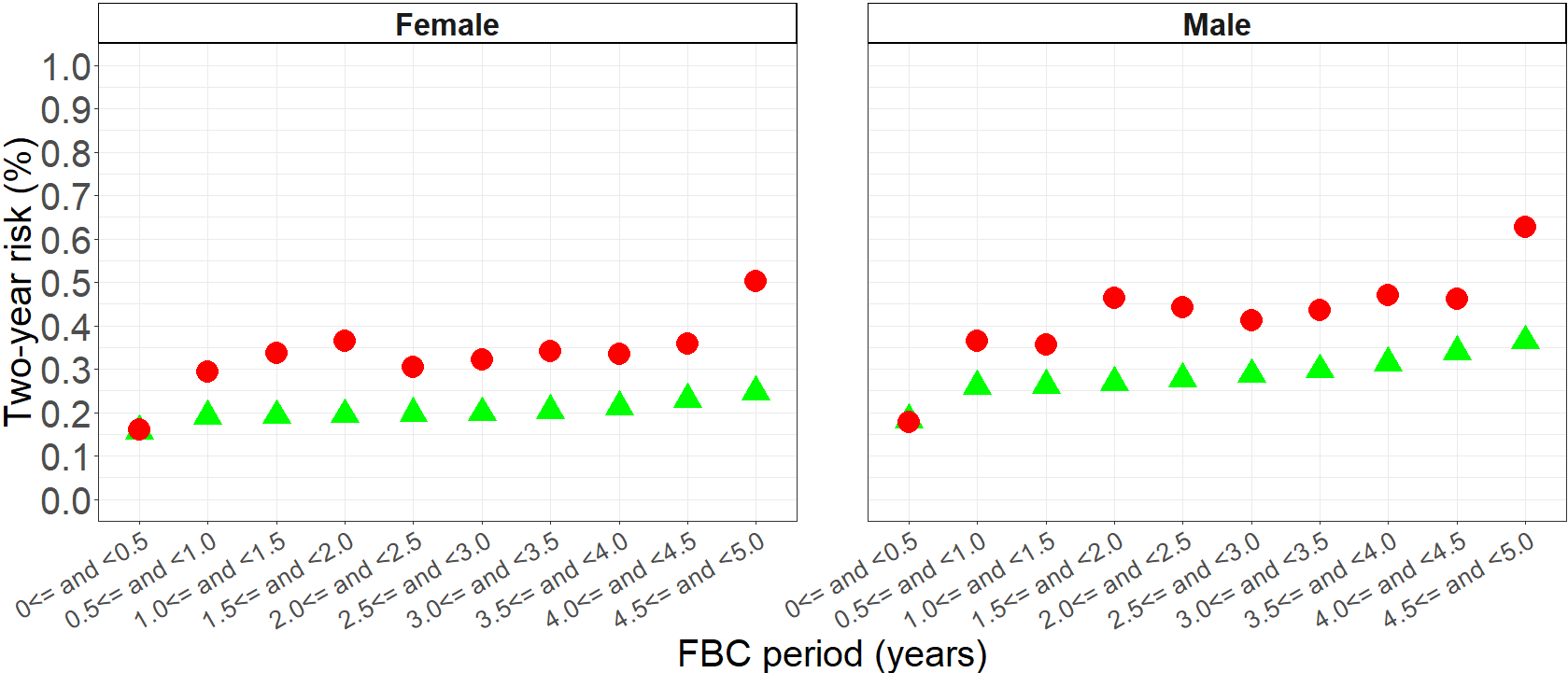


Abbreviations: KM=Kaplan-Meier

# Table S2: Diagnostic accuracy (95% CI) measures for males

| **Risk percentile** | **Risk threshold (%)** | **Sensitivity (%)** | **Specificity (%)** | **PPV (%)** | **NPV (%)** |
| --- | --- | --- | --- | --- | --- |
| 1 | 0.0110 | 99.95 (99.95-99.95) | 1.00 (0.99-1.01) | 0.43 (0.42-0.44) | 99.98 (99.98-99.98) |
| 5 | 0.0152 | 99.73 (99.72-99.74) | 5.02 (4.99-5.04) | 0.45 (0.44-0.45) | 99.98 (99.98-99.98) |
| 10 | 0.0215 | 99.36 (99.35-99.37) | 10.04 (10.00-10.07) | 0.47 (0.46-0.48) | 99.97 (99.97-99.97) |
| 15 | 0.0298 | 98.79 (98.78-98.80) | 15.06 (15.02-15.10) | 0.49 (0.49-0.50) | 99.97 (99.96-99.97) |
| 20 | 0.0406 | 98.15 (98.13-98.16) | 20.07 (20.03-20.12) | 0.52 (0.51-0.53) | 99.96 (99.96-99.96) |
| 25 | 0.0542 | 97.19 (97.17-97.21) | 25.09 (25.05-25.14) | 0.55 (0.54-0.56) | 99.95 (99.95-99.95) |
| 30 | 0.0713 | 95.97 (95.95-95.99) | 30.11 (30.06-30.16) | 0.58 (0.57-0.59) | 99.94 (99.94-99.95) |
| 35 | 0.0929 | 94.29 (94.27-94.32) | 35.12 (35.07-35.18) | 0.62 (0.61-0.63) | 99.93 (99.93-99.93) |
| 40 | 0.1201 | 92.08 (92.05-92.11) | 40.14 (40.08-40.19) | 0.65 (0.64-0.66) | 99.92 (99.91-99.92) |
| 45 | 0.1533 | 89.10 (89.06-89.14) | 45.14 (45.09-45.20) | 0.69 (0.68-0.70) | 99.90 (99.89-99.90) |
| 50 | 0.1920 | 85.31 (85.27-85.35) | 50.15 (50.09-50.21) | 0.73 (0.72-0.74) | 99.88 (99.87-99.88) |
| 55 | 0.2344 | 80.59 (80.55-80.64) | 55.15 (55.09-55.21) | 0.76 (0.75-0.77) | 99.85 (99.85-99.85) |
| 60 | 0.2777 | 74.89 (74.84-74.94) | 60.15 (60.09-60.20) | 0.80 (0.79-0.81) | 99.82 (99.82-99.83) |
| 65 | 0.3195 | 68.22 (68.17-68.28) | 65.14 (65.09-65.20) | 0.83 (0.82-0.84) | 99.79 (99.79-99.80) |
| 70 | 0.3577 | 60.75 (60.69-60.80) | 70.13 (70.08-70.18) | 0.86 (0.85-0.87) | 99.76 (99.76-99.77) |
| 75 | 0.3926 | 52.60 (52.54-52.66) | 75.12 (75.07-75.17) | 0.89 (0.88-0.91) | 99.73 (99.73-99.74) |
| 80 | 0.4264 | 43.54 (43.49-43.60) | 80.10 (80.05-80.15) | 0.93 (0.92-0.94) | 99.70 (99.69-99.71) |
| 85 | 0.4626 | 34.23 (34.17-34.28) | 85.08 (85.04-85.12) | 0.97 (0.96-0.98) | 99.67 (99.66-99.68) |
| 90 | 0.5079 | 24.30 (24.26-24.35) | 90.06 (90.03-90.09) | 1.03 (1.02-1.05) | 99.64 (99.64-99.65) |
| 95 | 0.5839 | 13.63 (13.59-13.67) | 95.04 (95.01-95.06) | 1.16 (1.15-1.17) | 99.61 (99.61-99.62) |
| 99 | 0.7810 | 3.56 (3.54-3.58) | 99.01 (99.00-99.02) | 1.52 (1.50-1.53) | 99.59 (99.58-99.59) |

# Table S3: Diagnostic accuracy (95% CI) measures for females

| **Risk percentile** | **Risk threshold (%)** | **Sensitivity (%)** | **Specificity (%)** | **PPV (%)** | **NPV (%)** |
| --- | --- | --- | --- | --- | --- |
| 1 | 0.0084 | 99.88 (99.88-99.89) | 1.00 (0.99-1.01) | 0.34 (0.33-0.34) | 99.96 (99.96-99.96) |
| 5 | 0.0120 | 99.48 (99.47-99.49) | 5.01 (4.99-5.03) | 0.35 (0.34-0.36) | 99.97 (99.96-99.97) |
| 10 | 0.0168 | 98.88 (98.87-98.89) | 10.02 (9.99-10.06) | 0.37 (0.36-0.37) | 99.96 (99.96-99.96) |
| 15 | 0.0228 | 98.18 (98.17-98.20) | 15.04 (15.01-15.08) | 0.39 (0.38-0.39) | 99.96 (99.96-99.96) |
| 20 | 0.0304 | 97.30 (97.29-97.32) | 20.06 (20.02-20.10) | 0.41 (0.40-0.41) | 99.95 (99.95-99.96) |
| 25 | 0.0400 | 96.35 (96.33-96.37) | 25.07 (25.03-25.12) | 0.43 (0.42-0.44) | 99.95 (99.95-99.95) |
| 30 | 0.0521 | 94.94 (94.92-94.96) | 30.08 (30.04-30.13) | 0.45 (0.45-0.46) | 99.94 (99.94-99.95) |
| 35 | 0.0676 | 93.58 (93.55-93.60) | 35.10 (35.05-35.14) | 0.48 (0.48-0.49) | 99.94 (99.94-99.94) |
| 40 | 0.0878 | 91.47 (91.44-91.50) | 40.11 (40.05-40.16) | 0.51 (0.50-0.52) | 99.93 (99.93-99.93) |
| 45 | 0.1133 | 88.95 (88.92-88.98) | 45.11 (45.06-45.17) | 0.54 (0.53-0.55) | 99.92 (99.91-99.92) |
| 50 | 0.1434 | 85.96 (85.93-86.00) | 50.12 (50.07-50.17) | 0.58 (0.57-0.58) | 99.91 (99.90-99.91) |
| 55 | 0.1750 | 82.08 (82.04-82.12) | 55.12 (55.07-55.18) | 0.61 (0.60-0.62) | 99.89 (99.89-99.89) |
| 60 | 0.2057 | 76.94 (76.90-76.98) | 60.12 (60.07-60.17) | 0.64 (0.64-0.65) | 99.87 (99.87-99.87) |
| 65 | 0.2342 | 70.99 (70.94-71.03) | 65.12 (65.07-65.17) | 0.68 (0.67-0.69) | 99.85 (99.85-99.85) |
| 70 | 0.2605 | 63.93 (63.88-63.98) | 70.11 (70.07-70.16) | 0.71 (0.71-0.72) | 99.83 (99.82-99.83) |
| 75 | 0.2857 | 56.65 (56.59-56.70) | 75.11 (75.06-75.15) | 0.76 (0.75-0.77) | 99.81 (99.80-99.81) |
| 80 | 0.3122 | 48.29 (48.24-48.34) | 80.09 (80.05-80.14) | 0.81 (0.80-0.82) | 99.78 (99.78-99.79) |
| 85 | 0.3429 | 38.87 (38.82-38.92) | 85.08 (85.04-85.12) | 0.87 (0.86-0.88) | 99.76 (99.75-99.76) |
| 90 | 0.3840 | 28.93 (28.88-28.98) | 90.06 (90.03-90.09) | 0.97 (0.96-0.98) | 99.74 (99.73-99.74) |
| 95 | 0.4579 | 17.06 (17.02-17.10) | 95.04 (95.02-95.06) | 1.14 (1.13-1.15) | 99.71 (99.70-99.71) |
| 99 | 0.6872 | 5.03 (5.00-5.05) | 99.01 (99.00-99.02) | 1.68 (1.67-1.70) | 99.68 (99.67-99.68) |

# Table S4: Summary of cancer diagnosis by age group

| **Cancer (n (%))** | **Main analysis**  **(NCRAS cancers)** | | **Sensivity analysis**  **(NCRAS+CPRD+HES+ONS cancers)** | |
| --- | --- | --- | --- | --- |
|  | **Males** | **Females** | **Males** | **Females** |
| Overall | 12,578 (0.4%) | 11,939 (0.3%) | 13,423 (0.5%) | 12,919 (0.4%) |
|  |  |  |  |  |
| Age (years) at current test |  |  |  |  |
| Age 18-39 |  |  |  |  |
| Age 40-49 | 328 (0.1%) | 446 (0.1%) | 375 (0.1%) | 495 (0.1%) |
| Age 50-59 | 1,265 (0.2%) | 1,018 (0.1%) | 1,352 (0.2%) | 1,125 (0.1%) |
| Age 60-69 | 3,357 (0.5%) | 2,191 (0.3%) | 3,553 (0.6%) | 2,341 (0.3%) |
| Age 70-79 | 4,628 (0.9%) | 4,006 (0.7%) | 4,920 (0.9%) | 4,273 (0.7%) |
| Age 80-89 | 2,723 (0.9%) | 3,704 (0.8%) | 2,919 (0.9%) | 4,018 (0.8%) |
| Age 90+ | 277 (0.6%) | 574 (0.4%) | 304 (0.6%) | 667 (0.5%) |

# Figure S11: Net benefit plots


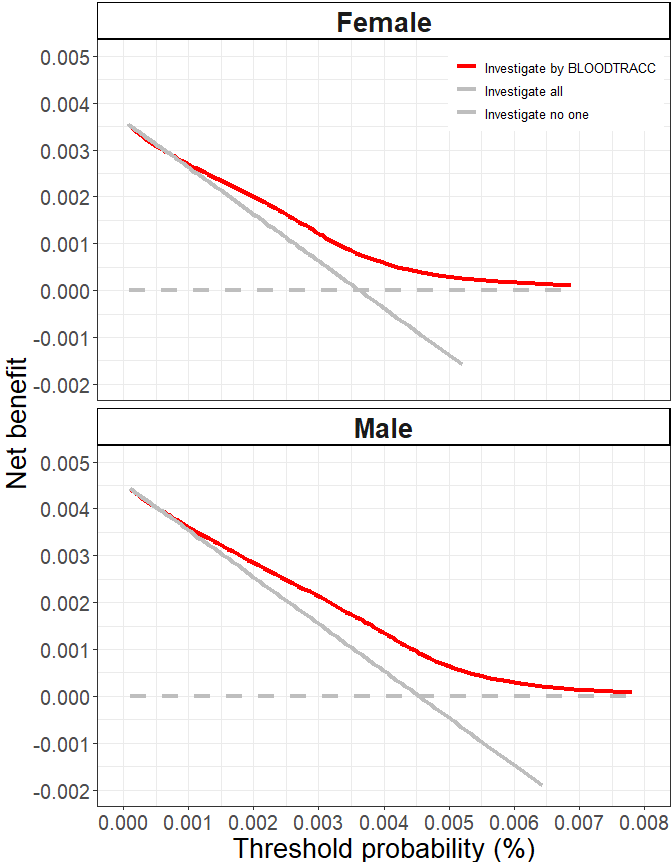


# Figure S12: Calibration plots for the BLOODTRACC models – sensitivity analysis (NCRAS+CPRD+HES+ONS cancers)


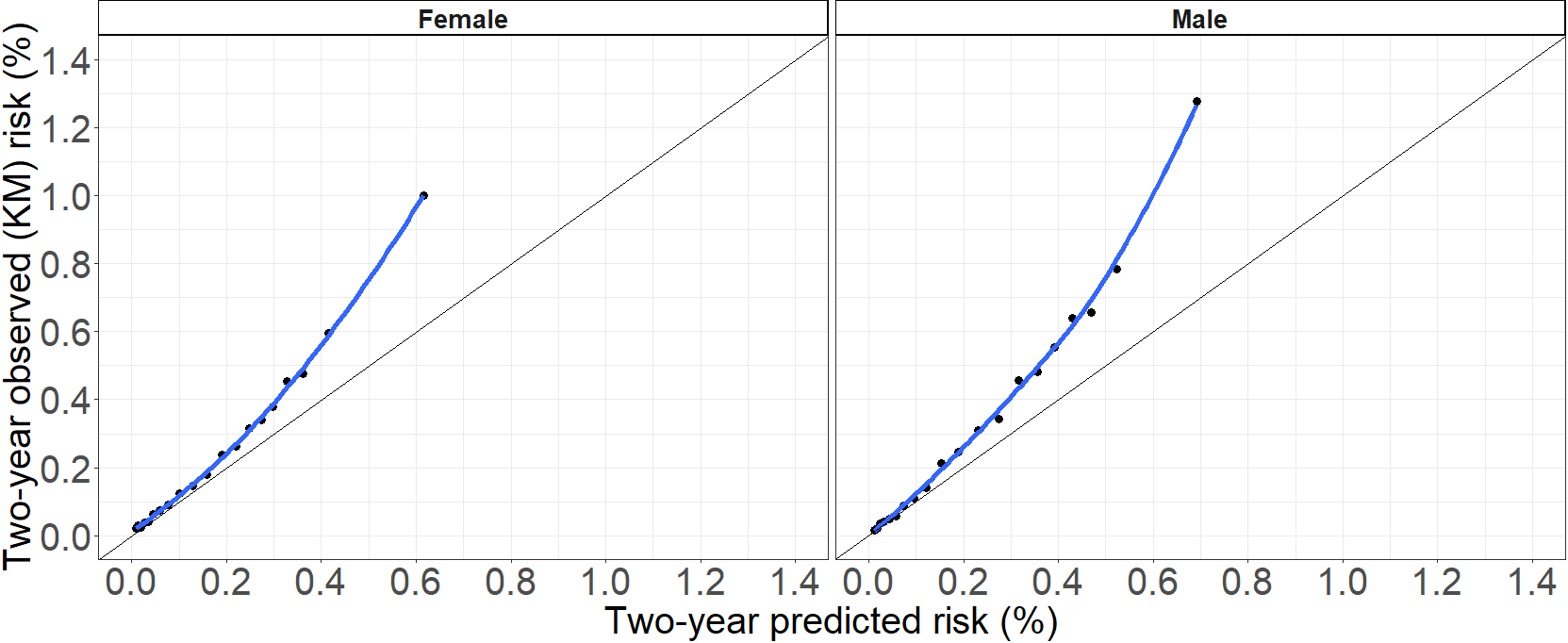

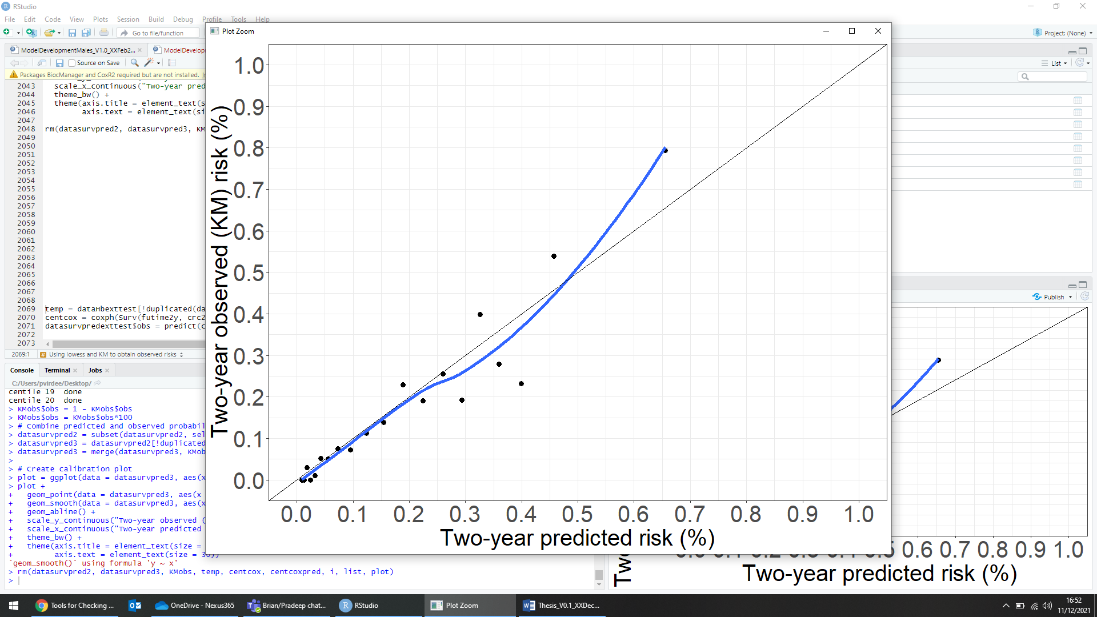


Risk group


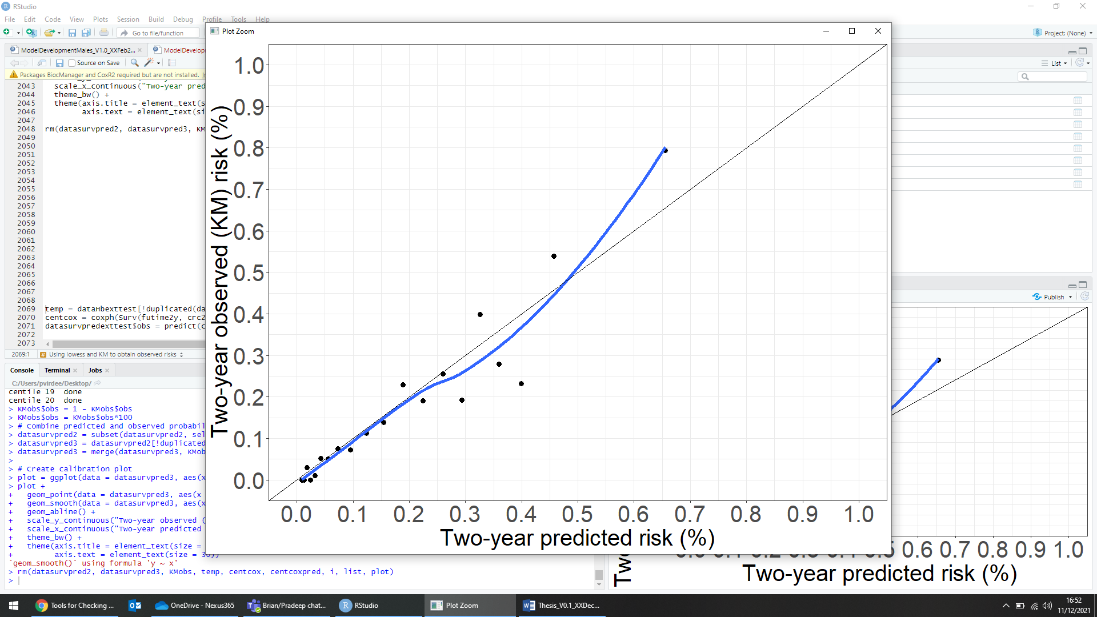


LOWESS


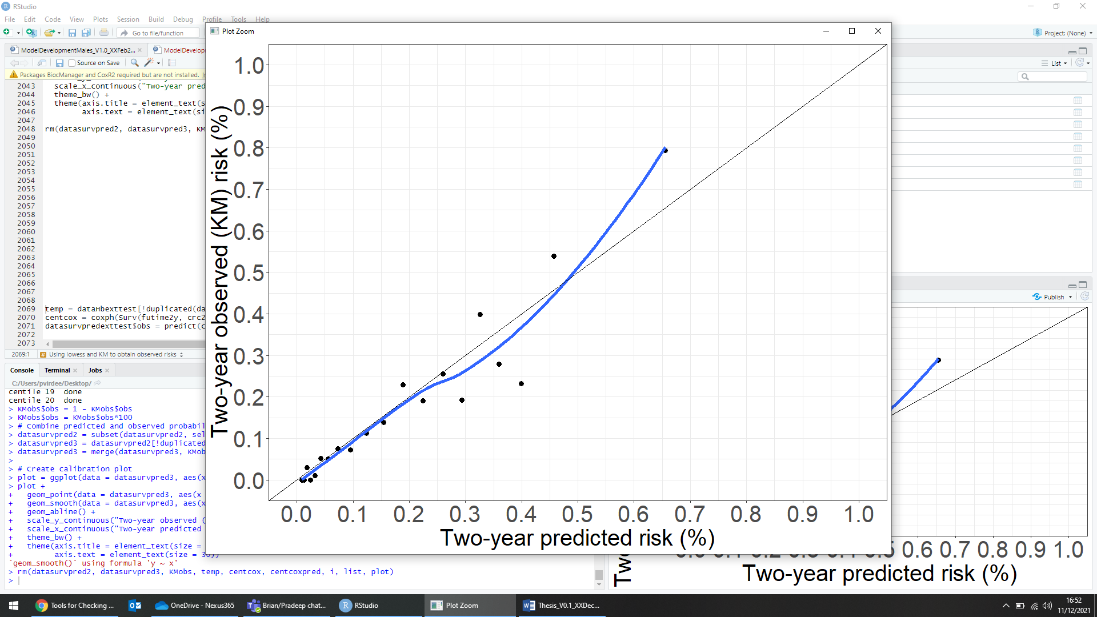


Reference

# Table S5: Summary of cancer diagnosis by study design and age group

| **Cancer (n (%))** | **Males** | | **Females** | |
| --- | --- | --- | --- | --- |
|  | **Main analysis (hybrid)** | **Sensitivity analysis (cohort)** | **Main analysis (hybrid)** | **Sensitivity analysis (cohort)** |
| Overall | 12,578 (0.4%) | 18,876 (0.4%) | 11,939 (0.3%) | 17,501 (0.3%) |
|  |  |  |  |  |
| Age (years) at current test |  |  |  |  |
| Age 40-49 | 328 (0.1%) | 795 (0.1%) | 375 (0.1%) | 724 (0.1%) |
| Age 50-59 | 1,265 (0.2%) | 2,211 (0.3%) | 1,352 (0.2%) | 1,617 (0.2%) |
| Age 60-69 | 3,357 (0.5%) | 4,217 (0.6%) | 3,553 (0.6%) | 2,614 (0.4%) |
| Age 70-79 | 4,628 (0.9%) | 5,703 (0.9%) | 4,920 (0.9%) | 4,470 (0.7%) |
| Age 80-89 | 2,723 (0.9%) | 4,668 (1.1%) | 2,919 (0.9%) | 5,766 (0.9%) |
| Age 90+ | 277 (0.6%) | 893 (0.9%) | 304 (0.6%) | 1,886 (0.8%) |
